# Outcome of one hundred consecutive ICSI attempts using patient operated home sonography for monitoring follicular growth

**Published:** 2016-09

**Authors:** J Gerris, F Vandekerckhove, P De Sutter

**Affiliations:** Women’s Clinic, University Hospital Ghent, De Pintelaan 185, 9000 Ghent, Belgium

**Keywords:** ART, follicular growth monitoring, home sonography, ovarian stimulation, tele-medicine, patient empowerment

## Abstract

**Objective:**

cohort study describing clinical and laboratory outcomes in ICSI patients using self-operated endovaginal tele-monitoring (SOET).

**Setting:**

University department of reproductive medicine.

**Patients:**

78 patients undergoing 100 consecutive ICSI attempts.

**Interventions:**

patients’ recorded vaginal sonograms and sent recordings using the cloud based Sonaura device to the care provider who procured responses, avoiding hospital visits.

**Main Outcome Measures:**

Number of cycles without hospital visit, laboratory and clinical variables, transportation avoided.

**Results:**

In 100 attempts, only one patient missed her follicles, 9 hospital visits occurred for circumstantial reasons and 90 attempts were completed without any hospital visit between initiation and puncture. Mean number ± 2SD was for oocytes 11.7±6.6, metaphase-II oocytes 8.5±5.4, 2PN zygotes 5.5±3.7, good day-5 blastocysts 2.4±2.4, embryos transferred 1.0±0.7 and embryos frozen 1.3±2.2. Percentages of total (+HCG), clinical (cardiac activity) and on-going pregnancies (>20 weeks) were 40%, 35% and 29% per started cycle and 48.8%, 42.7% and 35.4% per embryo transfer.

**Conclusions:**

Using SOET, 90% of ICSI (and IVF) patients can avoid visits to care providers for making sonograms. Results were similar as in patients with a similar profile using traditional monitoring. In appropriate patients, SOET is an efficient, safe and patient-friendly alternative for ovarian stimulation monitoring in IVF/ICSI programmes.

## Introduction

Vaginal sonographic follow-up of follicular growth is essential for in-vitro fertilization (IVF) or intracytoplasmic injection (ICSI) treatment. It cannot be dispensed with: counting and measuring growing follicles allows optimal dosage of hormones and correct timing of administration of the ovulation trigger. Early detection of ovarian hyperstimulation allows to freeze embryos and transfer frozen-thawed embryos later. Patients generally come for sonograms to the centre where oocyte retrieval, embryo culture and transfer take place, or see a gynaecologist, sonographer, general practitioner, midwife or nurse, elsewhere, usually implying frequent ravel. We have explored the feasibility and the outcome of self-operated endo- vaginal tele-monitoring (SOET) where patients perform sonograms anytime anywhere (Gerris 2009, 2010). In a randomized controlled trial, we compared the clinical, laboratory, subjective and health-economic outcome of SOET versus home monitoring (Gerris et al., 2014), showed non- inferiority for all variables studied. The server-based industrial prototype we used in that study has been replaced with a user-friendly, secure cloud-based solution, called Sonaura. We now report on outcome data of this method used in one hundred consecutive ICSI cycles.

## Subjects and methods

### Selection of patients

Between August 2014 and November 2015, a total of 78 ICSI patients from the Department of Reproductive Medicine of Ghent University Hospital, used the Sonaura system. Home monitoring using Sonaura was suggested especially to patients living at great distance or suffering systematic traffic jams.

Over a 14 months period, 78 different patients chose to be monitored using the Sonaura system (Sonaura LLC, Fort Collis, Colorado, USA). Indications for treatment were all male: oligoasthenoteratospermia (n=66), obstructive (n=9) or non-obstructive (n=25) azoospermia. Since all these indications require ICSI treatment, implying oocyte denudation, the number of metaphase-II oocytes was the primary outcome variable. This outcome variable cannot be assessed in regular IVF patients, who may of course also benefit from home monitoring. Back-up in-house sonography was available at all times. Patients were counselled regarding the possibility of poor response and hyperstimulation. There was no systematic serum estradiol measurement.

Routine pelvic sonography using a high-end instrument is mandatory before considering treatment in order to establish normal pelvic anatomy. Subserous myomata and PCO are no contra-indications for performing SOET. Submucous myomata, endometriosis cysts >3cm, an untreated septum or other congenital or acquired anatomical anomalies, interfering with implantation but also with sonographic follow-up are contra- indications for treatment, hence for SOET.

### Teaching of patients

Patients are shown the introductory movie at www.mysonaura.com, in order to give a first idea of the method. Proper teaching is only needed when the system is used for the first time (n=78), not in repeat users (n=22). A vaginal sonogram is performed, using a high-end instrument for didactic reasons, either after suppression with an oral contraceptive, or in a natural cycle. The probe is applied with gel and condom before it is introduced in the vagina. The recording sequence is: 30 seconds right ovary, 15 seconds uterus, 30 seconds left ovary. The bladder should be slightly filled for teaching, not for recording. The pelvis should be normal with a normal location of the ovaries. The black streaks of the pulsating iliac artery and the pulseless veins are identified, allowing to visualize the adjoining slightly darker greyish ovaries and perform an antral follicle count. Ovarian veins may be obvious and are pointed out to the patient as being no follicles. Usually the endometrium is visible although not with the triple line image.

The movements of the probe in all directions (laterolateral, anteroposterior and rotating) are demonstrated when searching for the resting ovaries.

It is pointed out that SOET is not a goal but a facilitating tool. Should either the doctor or the patient desire so, a professional sonogram is available. It is reminded that first recordings usually are neither easy nor perfect, but become so with the growing of the follicles and autodidactic experience of the patient. First sonograms were requested on day 5 of gonadotropin injection. It is pointed out that once the ovaries have been found, they will be in the same area on subsequent days.

Written instructions explaining each step of the procedure are given to the patient, who can use e-mail, SMS and telephone for all subsequent questions, although she is encouraged to do so via the system.

The uterus is outlined first and compared to a greyish pear-like organ, containing a central banana- like image, the endometrium, which is going to disappear with the withdrawal bleeding and then steadily comes back ending up showing the triple- line image. It is also mentioned to the patient that images of the uterus are not very important, because gonadotrophin dosage is not dependent on endometrial thickness but on ovarian follicle growth. Recording the uterus allows to distinguish the left from the right ovary.

### The Sonaura device

**Fig. 1:**
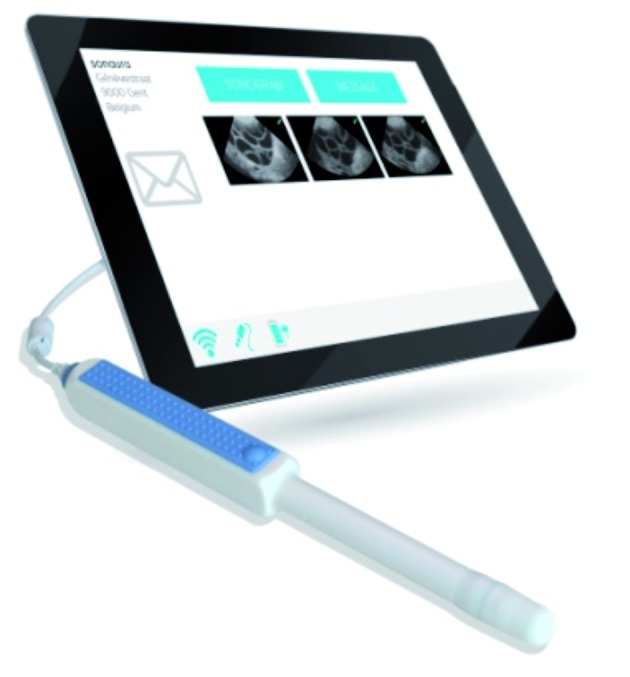
— The Sonaura system consists of a dedicated tablet connected to a vaginal probe using USB technology.

The sonographic device used (Sonaura, Fort Collins, Colorado, USA) (Fig. 1) consists of a patient part and a care provider part.

The patient part consists of a dedicated tablet, connectable to a FDA and CE compatible vaginal probe (Interson, Pleasanton, California, USA), delivered in a suitcase with gel, condoms and the written instructions. In between cycles, the probe is sanitized using a specific sporicidal and disinfectant foam (Tristel Duo, Tristel Solutions Limited, UK) based on chlorine dioxide (Casalegno et al., 2012; Ma et al., 2012). All cleansing within a cycle is done by dry cloth or absorptive paper. Images belonging to one particular active cycle are kept on the tablet until it is returned at the time of oocyte retrieval. They are wiped as soon as the cycle is ended by the care provider which makes the tablet available for another cycle.

The care provider part consists of specific software that can be accessed anytime from any personal computer through a user specific password. Access can be customized per doctor or per centre and contains a home page, a list of all patients, of patients presently in treatment, and of currently active cycles. When creating a new cycle, a cycle specific password and entry code are generated and automatically sent by mail to the patient, who needs them for each recording. When a new recording comes in, the care provider is notified by mail on his mobile phone. All images are kept securely and permanently in the cloud, where they can always be accessed. For measurement, recordings can be stopped and replayed as often as needed. All follicles are measured in their two largest perpendicular diameters. When measurement is completed, a note is entered in the patient’s communication box comprising a brief description of the stimulation status, dosage instructions, the time when the next sonogram is expected and, if needed, suggestions to improve image recording or some supportive words, and then sent to the patient, who also receives a mail indicating that the response is available on the tablet. All videos are stored in the cloud from where they can be retrieved again anytime and anywhere.

### Performing the vaginal sonograms

Patients usually perform sonograms at home, but can do so wherever they are provided they have access to Wi-Fi. Recordings were received, analysed and responded to by the care provider on different locations all over the world, although the idea is to make these interactions fit within the time frame of a regular ART clinic.

Women are requested to scan the ovaries and the uterus in a fixed order: first the right ovary, then the uterus and finally the left ovary. These recordings are sent as one uninterrupted recording. Each recording is preceded by a scanning period, during which the patient navigates the probe allowing her to create a mental picture of the scenario she is about to record. This may take some time and varies between 5 and 30 minutes. Women usually scanned and recorded themselves, requiring help from their partners when it was difficult. After recording, the patient has access to a communication box where she can write down a message or ask questions.

Patients were instructed to visualize the majority if not all of the largest follicles in such a way that the largest diameter and the diameter perpendicular to it come into view. Recording starts with the probe pointed towards the largest follicle in its largest diameter. A major challenge resides in the first two or three recordings when follicles are still small. Finding their way into the three-dimensional pelvis using a two-dimensional instrument makes the woman go through an “auto-didactic” phase that can be very short or take several days. Almost all patients succeeded in making very clear recordings, especially once follicles are >15mm and near the end of stimulation.

### Stimulation protocol and follow-up

Patients were suppressed using an oral contraceptive after which an individualized short agonist scheme was initiated consisting of 0.1mg of decapeptyl (DecapeptylR, Ferring, Denmark) and a standard starting dose of 150IU of Gonal-f (follitropin alfa, Merck Serono, Germany or Menopur, Ferring, Denmark) on the third day of agonist administration. Alternatively, patients with an expected high response could also start in a natural cycle and were given gonadotrophins together with an antagonist (cetrorelixR, CetrotideR, Merck Serono, Germany). Adaptations of the daily dosage were carried on the basis of sonographic images sent by the patient exclusively. The first images were requested on the fifth day of gonadotrophin stimulation. Within hours, the patients received a response indicating the further gonadotrophin dosage and when the next sonogram was expected. When the last sonogram was obtained, detailed instruction on the administration of 5000 IU of human chorionic gonadotrophin (PregnylR) was given through the Sonaura system, 36 hours prior to oocyte retrieval, taking into account that timing of HCG injection is still a matter of debate (Chen et al., 2014). The patient was always contacted by telephone the next day to confirm these instructions.

All these cycles were followed up by sonography only. Systematic serum estradiol determinations do not improve the prevention of OHSS nor to the chance for pregnancy (Kwan et al., 2014; Martins et al., 2014).

### Oocyte retrieval, laboratory phase, embryo transfer and luteal phase

Oocyte retrievals, oocyte handling and fertilization were conducted using standard equipment and procedures. Transfers were performed on day 5 using Cook transfer catheters. The luteal phase was supported using oral micronized progesterone (3 x 200mg/day).

We recorded the total pregnancy rate, i.e. all cycles with at least two HCG rising values > 5mIU/ ml or one value >50mIU/ml. The clinical pregnancy rate was defined by either seeing a least one amniotic sac or an HCG >2000 mIU/ml and an on-going pregnancy is defined as a pregnancy >20 weeks. In a biochemical conception, serum HCG values always remained <1000mIU/ml and an amniotic sac was never visible.

## Results

Of a total of 78 different patients, 62 patients went through one, 11 through two, 4 through three and one patient through 4 ICSI attempts using Sonaura. Mean age of the women was 35,2±3,8 (range: 26- 44) years of age. A mean total dose of 2857±1349 IU of gonadotrophins was used (range: 412-6750 IU).

A total of 471 home sonograms were performed and analysed (mean=4,71±1,48/attempt; range: 2-9). Ninety cycles were conducted without any in situ sonographic control between the start of the treatment and the moment of oocyte retrieval. Table I describes reasons why in 10 cycles at least one in-situ sonogram was performed.

**Table I:** — Reasons for performing at least one on-site sonogram in ICSI patients followed using Sonaura.

**Case**	**Reason why in-situ sonogram was performed**	**Frequency of event**
1	Confirmation of correctly recorded total absence of response resulting in cancellation	1x
2	Confirmation of a very poor but correctly recorded response (1 follicles and twice 2 follicles).	3x
3	Confirmation of a very poor response (4 follicles) not correctly recorded by the patient.	1x
4	Confirmation of very slow response, in situ sonogram after 5 home sonograms, followed by 4 more home sonograms, resulting in 18 follicles and pregnancy	1x
5	Very slow stimulation requiring patient to come (twice) for additional drugs at our pharmacy	2x
6	Patient happened to be in Ghent for other reasons and requested sonogram but correct recordings	2x
		10 cases

We had the patient come to the clinic for a sonogram whenever on the 12^th^ day of gonadotropin administration no reliable recording was available yet, in order to distinguish between a true poor or slow response and a true failure to detect follicles. Another reason to have the patient come in were sonographic signs of ovarian hyperstimulation, when serum blood testing for estradiol was warranted. In our own series, six cases of threatening hyperstimulation were detected but all six chose to continue at home and have a puncture and an all- freeze cycle. Three of them became pregnant using frozen/thawed embryos afterwards.

Table II describes the main laboratory outcome data in 100 ICSI attempts using Sonaura. Conception results are expressed as cumulative pregnancy rate, truly reflecting the cumulative potential for a first on-going pregnancy from these home-followed cycles.

**Table II:** — Main laboratory outcome data in 100 ICSI attempts using Sonaura.

**Variable**	**Average ± 2SD**	**Range**
N oocytes retrieved	11,5 ± 6.5	1 - 30
N mature oocytes used for ICSI	8,5 ± 5.4	1 - 27
N 2PN-zygotes	5,4 ± 3,7	0 - 17
N usable day-5 embryos	2,4 ± 2,4	0 - 12
N embryos transferred in all patients	1,0 ± 0,7	0 – 2
N embryos transferred in patients with ET	1,32 ± 0,47	1 - 2
N embryos frozen in all patients	1,3 ± 2,2	0 – 12
N embryos frozen in patients with ET	1,39 ± 2,02	1 - 12

In 24 attempts, there was no embryo transfer, in six of which transfer was postponed due to a risk for ovarian hyperstimulation syndrome. Hence, there were 18 attempts in which no viable (either fresh or frozen) embryo was available. Pregnancy rates can thus be calculated both on the basis of all attempts started (n=100 minus one cancellation) and on the basis of transfers (n=82).

There were three on-going twins (both delivered) as well as two twin miscarriages. There were two cases of severe ovarian hyperstimulation syndrome, both of whom had an on-going pregnancy (one singleton, one twin). There were 29 on-going pregnancies, 6 clinical miscarriages, 5 biochemical conceptions and 1 pregnancy of unknown location.

In total, there were 40 cases with a positive serum HCG value. The total, clinical and on-going pregnancy rates per started cycle were 40%, 35% and 29%. In 18% of cycles, there was no embryo transfer. On a per transfer basis these figures are 48.8%, 42.7% and 35.4%. There were 29 on- going pregnancies in 78 different women = 37% of patients obtained an on-going pregnancy until now (Table III).

**Table III:** — Conception results from 100 consecutive ICSI cycles followed using Sonaura.

**Outcome measure**	**Average ± 2SD**
N of started cycles	100
Cancellations (no egg retrieval)	1 (1%)
N of oocyte retrievals	99 (99%)
N of transfers	82/99 (82.8%)
HCG/started cycle	40/100 (40%)
HCG/oocyte retrieval	40/99 (40.4%)
HCG/transfer	40/82 (48.8%)
Clinical pregnancies/started cycle	35/11 (35%)
Clinical pregnancies/egg retrieval	35/99 (35%)
Clinical pregnancies/transfer	35/82 (42.7%)
On-going pregnancies/started cycle	29/100 (29%)
On-going pregnancies/egg retrieval	29/99 (29.3%)
On-going pregnancies/transfer	29/82 (35.4%)

The mean two-way distance per sonogram from home to the centre was 376±114kms. With 4.7 sonograms per attempt, this means an average of 1.732 kilometres of avoided transportation. At 0.3461€/km, this is an average saving of €600 per attempt.

There were no complications. The large majority of patients and their partners were very positive about the use of the Sonaura system.

## Discussion

The system derives its potential value from two simple points: 1. Clinical decisions do not differ when taken on the basis of a high-end machine at the centre versus when taken on the basis of images made by the patient herself and 2. There is a complete disjunction between place and time for both patient and care provider, making the process of follow-up much more flexible. SOET can be performed anywhere anytime, at both sides of the application (Gerris, 2015).

The laboratory and clinical results in this group of patients using SOET are comparable with those in similar groups of patients who undergo traditional follicular growth monitoring. In this group of older patients, a no-transfer incidence of 18% is not abnormal.

The ideal patient to use SOET is the woman who has gone through at least one complete ART cycle and is not averse to innovation. She knows what growing follicles look like. She has logistic problems in attending the clinic, or children to attend, or a very demanding job, or she lives far away. She confirms that she can introduce a vaginal probe herself. Yet, we have used the instrument in first-ever ICSI attempts as well. Most patients who have used the system a first time, request to use it again. Though normal responders are easiest to follow, we have also used the system in known poor responders (Ferraretti et al., 2011). The patient also needs to be at ease with a simple vaginal manipulation. Measurements and feedback to the patient being the physician’s responsibility, the patient only needs to procure quality to make measurements possible. We have now shifted first recordings to the seventh or eighth day. There are wide differences between patients. Some of them are able to send perfect ovarian recordings when follicles are even smaller than 10mm in diameter, whereas others do not find the follicles easily before the largest ones are >15mm in diameter.

In this time of technological innovation, patients also want to be actively involved in their treatment. The advantages of a purely technological character are: less time, less transportation, lower transportation costs, less stress, more involvement of the partner, more discretion, possibility to make the images during leisure time, creating a stronger bond between the woman and her partner, who is in 50% of the cases at the origin of the subfertility problem. SOET is not only a technological innovation but also a method of improved patient-physician interaction.

Users of the system may benefit from hands-on training and learning how to deal with tricky situations. It is not only useful to train doctors, but also midwives and nurses, who are able to use the system under physician supervision. Though conceived as a method for patient empowerment, in particular circumstances, general practitioners, nurses, midwives or gynaecologists may be the actual operators of the system sending images to a distant centre where decisions are taken. It allows patients to choose the centre where they want their biology to be performed.

For care providers, it implies a change of practice organization but it ends up in more freedom, more time to see new patients.

Although most patients are able to procure reliable images, SOET will never be a method for all patients. As experience grows, more patients may feel attracted. We also need fair reimbursement and to rethink practice organization.

Another question pertains to image quality. Pereira et al. (2016) have conducted a study showing a very high correlation between measurements performed by the patient and by a professional in varying clinical situations where follicles were counted and measured.

The final question is who are the winners? Surely the patient does not only stand central, she is also actively involved in her treatment, a fact which strongly determines patient satisfaction. Though at first sight part of the onus falls onto the treating physician’s shoulders, this form of tele-medicine can be applied in a very structured way. For ambitious centres, using SOET may help to enlarge their action radius and extend it to distant and thinly populated areas. The silent winners are definitely the employers, as half of the lower cost of SOET versus traditional monitoring comes from avoiding absence from work (Gerris et al., 2014). This is also important if the patient and/or the partner exert an independent profession.
